# Syndecan Binding Protein (SDCBP) Is Overexpressed in Estrogen Receptor Negative Breast Cancers, and Is a Potential Promoter for Tumor Proliferation

**DOI:** 10.1371/journal.pone.0060046

**Published:** 2013-03-22

**Authors:** Xiao-Long Qian, Ya-Qing Li, Bin Yu, Feng Gu, Fang-Fang Liu, Wei-Dong Li, Xin-Min Zhang, Li Fu

**Affiliations:** 1 Department of Breast Cancer Pathology and Research Laboratory, Tianjin Medical University Cancer Institute and Hospital, Tianjin, China; 2 Key Laboratory of Breast Cancer Prevention and Therapy, Tianjin Medical University, Ministry of Education, Tianjin, China; 3 Key Laboratory of Cancer Prevention and Therapy, Tianjin, China; 4 Department of Pathology and Laboratory Medicine, Temple University Hospital, Philadelphia, Pennsylvania, United States of America; 5 2011 Collaborative Innovation Center of Tianjin for Medical Epigenetics, Tianjin, China; 6 Tian Jin University of Traditional Chinese Medicine, Tianjin State Key Laboratory of Modern Chinese Medicine, Tianjin, China; Wayne State University School of Medicine, United States of America

## Abstract

**Background:**

Syndecan binding protein (SDCBP), an adapter protein containing PDZ domains, contributes to the tumorigenicity and metastasis of many malignant tumors, such as malignant melanoma. Our study aimed in revealing the expression profile of SDCBP in breast cancer (BCa) and its role in tumor cell proliferation, and then exploring its value in the targeted treatment of BCa.

**Methodology/Principal Findings:**

We first evaluated the SDCBP expression by immunohistochemistry in normal breast and BCa tissues. Then we explored the expression profile of SDCBP in different BCa cell lines. By constructing SDCBP-silenced BCa cell clones, we further assessed the effects of SDCBP suppression on tumor cells *in vitro* by cell culture and *in vivo* by tumorigenicity. SDCBP expression was detected in 80.6% (n = 160) of BCa tissues, in contrast to its expression in 13% (n = 23) of normal breast tissues (*P*<0.001). Among the tumors, the level of its expression was positively correlated with histological grade and tumor staging while negatively correlated with the estrogen receptor (ER) expression. Higher expression of SDCBP was also noted in ER-negative BCa cell lines. It was also identified that SDCBP expression was down-regulated in a dose-dependent mode by 17-β estradiol in estrogen-responsive MCF-7. Furthermore, SDCBP silence inhibited ER-negative tumor cell growth *in vivo* and *in vitro*. Cell cycle studies showed that SDCBP silence increased G1 cell population and resulted in related cell-cycle-regulator changes: up-regulation of p21 and p27 while down-regulation of cyclin E.

**Conclusion/Significance:**

Our results suggested that SDCBP played an important role in tumor growth of ER-negative BCas. In these tumors where the estrogen signaling pathway is not available, SDCBP probably contribute to tumor growth through an alternative signaling pathway by promoting tumor cells passing the G1/S checkpoint into the cell cycle. Suppression of SDCBP expression may have its potential to become a targeted therapy for ER-negative BCas.

## Introduction

Breast cancer (BCa) is a heterogeneous disease including multiple subgroups with different molecular signatures, clinical characteristics, and responses to therapies [1]. The estrogen receptor (ER) negative BCa, particular the triple negative breast cancer (TNBC) which refers to any BCa that does not express the genes for estrogen receptor (ER), progesterone receptor (PR) or Human Epidermal Growth Factor Receptor 2 (HER-2/neu), has more aggressive clinical course and less effective treatments due to lack of specific targets. Although chemotherapeutic regimens were developed for TNBCs, they are limited in numbers and do not meet the pre-specified criteria [2]. It becomes obvious that there is urgent need to seek new targets and to develop corresponding therapeutic reagents with high efficacy.

Syndecan binding protein (SDCBP), also known as “syntenin” and melanoma differentiation associated gene-9 (MDA-9), was initially identified as a molecule linking syndecan-mediated signaling to the cytoskeleton [3]. It is a PDZ-domain-containing molecule that has many interaction partners, and regulates transmembrane-receptor trafficking, tumor-cell metastasis and neuronal-synapse function [4]. Li et al found, when eukaryotic translation initiation factor 5A (eIF5A) interacted with SDCBP, the eIF5A-induced increase in p53 protein level was significantly inhibited. This insinuated that SDCBP might have a role in apoptosis inhibition [5]. SDCBP was also reported to be responsible for cell migration, invasion and pseudopodia formation, all of which are link to tumor metastasis [6], [7]. SDCBP may carry its role by affecting the cytoskeleton system, possibly through altering the known signaling pathway such as focal adhesion kinase (FAK), p38 mitogen-activated protein kinases, c-jun NH2-terminal kinase and nuclear factor kB [8], [9]. It was reported that activation of PKCα could induce expression of SDCBP, and then enhance the FN-induced assembly of integrin-β1 signaling complexes with FAK and c-Src, resulting in activation of FAK and its downstream pathways [7]. SDCBP may also directly interact with c-Src, and facilitate the formation of active FAK/c-Src signaling complexes which is important for regulating the migration machinery [8]. The role of SDCBP in the melanoma metastasis has been extensively studied [7]–[9]. Nevertheless, only a few studies on the role of SDCBP in the progression of BCa have been pursued so far. Koo et al. reported that the level of SDCBP expression correlated well with invasion and metastasis of BCa [6]. However, the expression profile of SDCBP has not been characterized in BCa, and the mechanism by which it involves in the proliferation of BCa cells has not been investigated yet. This study is undertaken to evaluate the expression profile of SDCBP in BCa and to explore its role in the tumor cell proliferation, and thus to assess its potential value in the targeted treatment of BCa.

## Materials and Methods

### Ethics statement

All human breast tissues were collected with written informed consent from patients prior to participation in the study. The protocols for collection and analysis of the samples were approved by the Institutional Review Board of the Tianjin Medical University Cancer Institute and Hospital, in accordance with the current revision of the Helsinki Declaration. The Institutional Animal Care and Use Committee of the Tianjin Medical University Cancer Institute and Hospital approved the use of animal models in this study in accordance with EU Directive 2010/63/EU for animal experiments. All surgeries were performed under sodium pentobarbital anesthesia, and all efforts were made to minimize suffering.

### Human breast specimens

183 cases of breast specimens obtained from patients who underwent surgical excision from January to March 2010 were retrieved from the archive of Department of Breast Cancer Pathology and Research Laboratory, Tianjin Medical University Cancer Institute and Hospital (China), which included 160 cases of primary BCa and 23 cases of normal breast tissue. Tissue sections were reviewed independently to confirm the diagnosis and the histological grade of BCa by two pathologists using the WHO criteria. All patients were females with an age range from 31 to 75 years. No radiation and/or chemotherapy were offered to any of the patients with BCa before surgery.

### Immunohistochemistry

Immunohistochemistry for SDCBP was performed on formalin-fixed paraffin-embedded tissue blocks in all the cases. Briefly, 5 µm tissue sections were deparaffinized and rehydrated with xylene and a series of grades of alcohol and antigen retrieval was carried out in 5 mM citrate buffer (pH 6.0) in an autoclave. Then endogenous peroxidase activity was inactivated by incubation in 3% H_2_O_2_ for 10 min. Sections were blocked and then incubated with primary antibody (supplier and dilution as indicated in Table S1) at 37°C for 2 h. Further procedures were performed using SP Immunohistochemistry Kit (Zhongshan Golden Bridge, Beijing, China) according to the manufacturer's instructions. Sections incubated with phosphate-buffered saline (PBS) only served as negative controls. Immunohistochemistry for ER and HER-2 was performed in the cases of BCa, and a standard procedure was used (Table S1).

ER and SDCBP positivity were determined by scoring the proportion of stained cells plus a measurement of intensity of the staining as in reference [10]. The expression level was determined using a modified scoring system by multiplying the intensity score (0 = negative; 1 = low; 2 = medium; 3 = high) with the percentage of the stained cells. In cases when different staining intensity scores were present in different areas, the sum of all intensity scores multiplied by the cell proportion with this intensity score was made. Such a scoring system gives a final score range from 0 to 300.

Cases were considered positive for SDCBP if cytoplasmic staining was present with a final score higher than 50, and the final score was classified as: 0∼50 negative, 51∼100 weakly positive, 101∼200 moderately positive and 201∼300 strongly positive. For ER, cases were considered positive if nuclear staining was present with a final score higher than 50, and the final score was classified in the same way as for SDCBP. The immunohistochemistry for HER-2 was based on identification of membrane staining of tumor cells and it was scored according to the ASCO/CAP Guideline. [11]. All the cases were evaluated by two pathologists independently and any discrepancy was resolved by a group discussion.

### Cell culture

Human breast cell lines were purchased from American Type Culture Collection, including ER-positive BCa cell lines MCF-7 and T47D, ER-negative BCa cells MDA-MB-231, Hs 578T and BT-549 and the fibrocystic disease epithelial cell MCF-10A. They were propagated and subcultured as recommended. Human embryonic kidney 293T cell line (293T) was obtained as a gift from Dr. Jin-Tang Dong (Nankai University, China), and it had been used previously [12].

### Estrogen-treatment of MCF-7 cells

To deplete the estrogen, MCF-7 cells were cultured in phenol red-free RPMI 1640 with 2.5% charcoal/dextran-treated FBS (Hyclone) for 24 h. Then 17-β estradiol (E2, Sigma) was added into the culture medium to a final concentration of 0, 1, 3 and 10 nM respectively and cells were cultured for another 24 h.

### Semi-quantitative reverse transcription-PCR (RT-PCR) and quantitative reverse transcription-PCR (qRT-PCR)

Total RNA were purified by TRIzol Reagent (Invitrogen Life Technologies). First-strand cDNA was generated using the First Strand Synthesis System (TOYOBO, Japan) according to manufacturer's instruction. Primers for semi-quantitative RT-PCR and qRT-PCR of SDCBP were listed in Table S2 and β-actin was used as the internal control. The cycling conditions of semi-quantitative PCR were identical to those described in literature [13]. The real-time qRT-PCR assay was performed using ABI PRISM 7000 Sequence Detection System (Applied Biosystems, Foster City, CA) as that in literature [13]. SDCBP mRNA expression levels were normalized against β-actin mRNA expression.

### Western Blot

Holoproteins in cell lysates were extracted, quantitated and immunoblotted as described in literature [14], except the usage of following antibodies: Anti- human SDCBP antibody (Abcam, Inc.); anti- β-actin and anti- cyclin E antibody (Santa Cruz Biotechnology, Inc.); antibody against phosphorylated Rb at Ser780 (phospo-Rb (S780)), and antibodies against the cyclin dependent kinase inhibitor p21^Cip1^ (p21) and p27^Kip1^ (p27), (Cell Signaling Technology, Inc.). For comparing the expression of SDCBP at protein level in different mammary epithelial cells, the relative immunoblot intensity of SDCBP/β-actin from triplicate repeats of each cell line was calculated and represented by mean ± standard deviation. SNK grouping test (*q* test) was used to compare the difference among these cells.

### Construction of SDCBP-silenced BCa cells

Candidate target sequences for short-hairpin RNA (shRNA) of SDCBP and for negative control shRNA were designed by Genepharma Co., Ltd (Shanghai, China), as shown in Table S3. They were all cloned into pGPU6/GFP/Neo shRNA expression vector. These constructs were transiently transfected into 293T cells by Lipofectamine 2000 (Invitrogen Life Technologies) according to manufacturer's protocol. Transfected cells were harvested and their holoproteins were western-blotted for SDCBP shRNA selection. The candidate SDCBP shRNA with the best RNA interference effect was selected and used in this study. MDA-MB-231 and BT-549 cells were transfected with the SDCBP shRNA and negative control shRNA as above. Cells were selected and cultured with appropriate medium containing 0.5 mg/ml of G418 (Sigma) for about 3 weeks before they were used for western-blotting analysis. One MDA-MB-231 and one BT-549 stable clones with maximal SDCBP down-regulation were selected and were named as MDA-MB-231-SDCBP shRNA and BT-549-SDCBP shRNA respectively. Negative control shRNA transfected stable clones were named as MDA-MB-231-Control shRNA and BT-549-Control shRNA respectively.

### Cell growth curve analysis (MTT proliferation assay)

MDA-MB-231 or BT-549 cells were seeded 1000 per well in 96-well plates and incubated overnight for cell adherence. The first day after seeding was defined as “Days 0”, and so on. Each day, media in the corresponding wells were replaced by 200 µl new media containing 0.5 mg/ml 3-(4, 5-dimethylthiazol-2-yl)-2, 5-diphenylte-trazolium bromide (MTT), and the cells were incubated for additional 4 hr at 37°C. Then 150 µl of dissolving reagent DMSO (Amresco, Inc.) was added to dissolve the formazan crystals. The absorbance was measured at a wavelength of 490 nm (*A_490_*) on a microplate reader (Bio-Rad). Wells without seeded cells were used as blank, and the reading of each well was corrected. Each experiment was repeated independently seven times. The mean of the seven repeats of *A_490_* corrected readings in each day was divided by that in “Days 0”, and the ratio (*A_490_* ratio) in each day was calculated accordingly. Linear regression analysis between “*A_490_* ratio” and “days” of both control and shRNA groups were performed using SPSS 13.0 software.

### Flow cytometric cell cycle analysis

Cell cycle of MDA-MB-231 or BT-549 was analyzed on a BD FACS Calibur flow cytometer as described in literature [14].

### *In vivo* tumorigenicity study

Female athymic BALB/c mice, 6 to 8 weeks old (Vital River Laboratories, China) were used to investigate the tumorigenicity of the SDCBP silence and the control cells. A total of 1×10^6^ MDA-MB-231-SDCBP shRNA cells at the exponential growth phase were suspended in 100 µl serum-free RPMI 1640 containing Matrigel (1∶1, vol/vol; BD Biosciences) and injected subcutaneously into the back of each of the 5 mice via 27-gauge needle, as the experimental group. The 5 mice in the control group were inoculated with MDA-MB-231-Control shRNA cells in same dosage and same procedure. The tumor size was measured once a week and the tumor volumes were calculated by the formula of Length×Width×Height×0.5236.

## Results

### SDCBP expression in breast tissues

**Figure 1:**
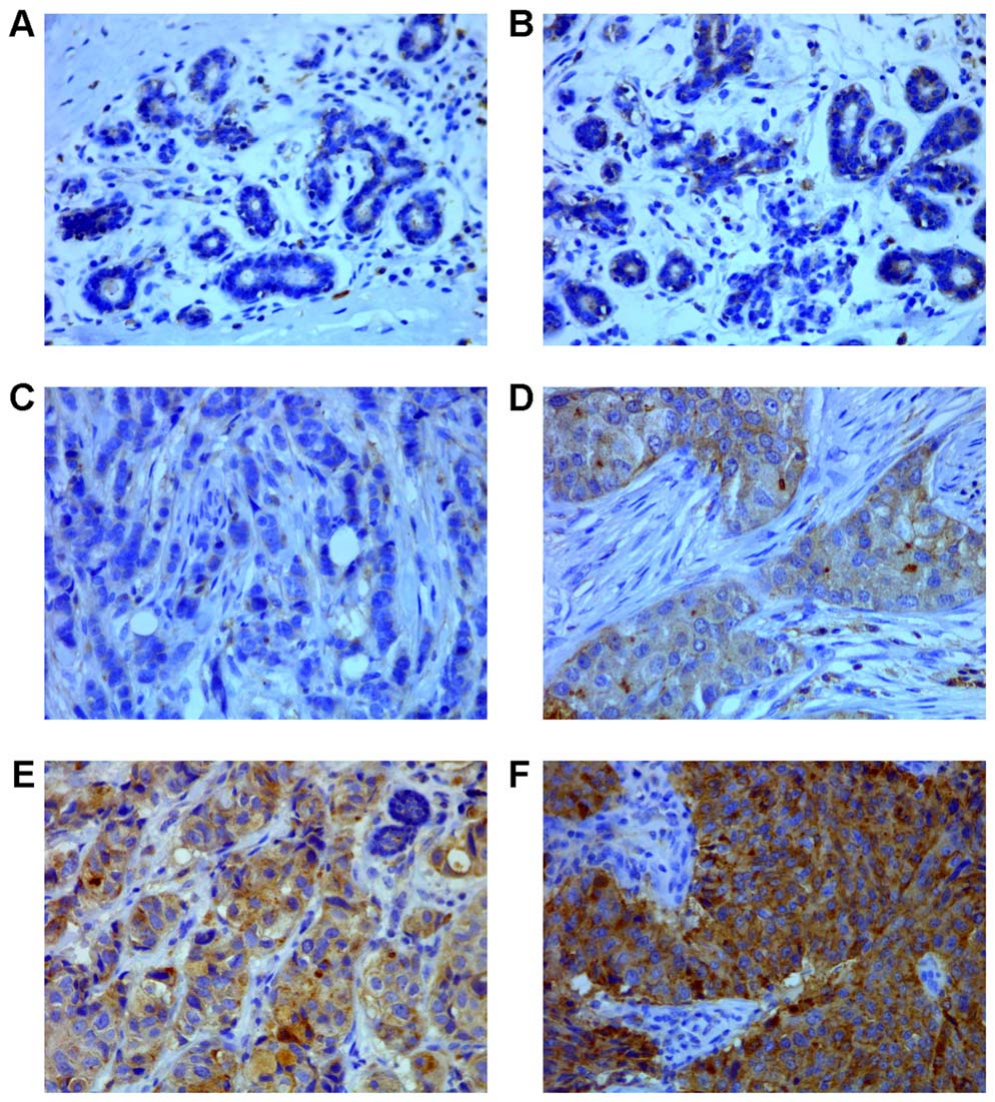
SDCBP expression in normal breast and breast cancer tissue. (**A**) Negative expression of syndecan binding protein (SDCBP) in normal breast tissue (×400). (**B**) Weak expression of SDCBP in normal breast tissue (×400). (**C**) Negative expression of SDCBP in breast cancer (BCa, ×400). (**D**) Weak expression of SDCBP in BCa (×400). (**E**) Moderate expression of SDCBP in BCa (×400). (**F**) Strong expression of SDCBP in BCa (×400).

SDCBP expression was identified immunohistochemically in 129 of 160 (80.6%) of BCas, while in 3 of 23 (13%) normal breast tissues where only weak staining was noted (*P*<0.001, calculated by Mann-Whitney U test; Table 1 and Figure 1). Among the BCas, 27.9% (17/61) of ER-negative tumors demonstrated strong cytoplasmic stain, while none of the ER strong-positive (0/45) tumors demonstrated strongly positive stain for SDCBP; a negative correlation between SDCBP expression and estrogen receptor (ER) status was also significantly established (R_S_ = −0.421, *P*<0.001; Table 2). It was also identified that SDCBP expression was positively correlated with tumour histological grading (R_S_ = 0.233, *P* = 0.003) and pTNM staging (R_S_ = 0.163, *P* = 0.04); however a positive association of SDCBP expression with tumor HER-2 overexpression was not significantly established (*P* = 0.316; Table 2). Additional findings of SDCBP expression in association with other pathological features of BCas were also shown in Table 2.

**Table 1:** Syndecan binding protein expression in normal breast and breast cancer tissue.

	Cases	Syndecan binding protein (%)				*P* value*
		Negative	Weak	Moderate	Strong	
Normal breast tissue	23	20(87.0)	3(13.0)	0(0.0)	0(0.0)	<0.001
Breast cancer tissue	160	31(19.4)	62(38.8)	42(26.3)	25(15.6)	

* , the *P* value was calculated by Mann–Whitney *U*-test.

**Table 2:** Syndecan binding protein expression and pathological features of breast cancers.

Pathological features	Cases	Syndecan binding protein (%)				*r*_s_	*P* value*
		Negative	Weak	Moderate	Strong		
Age (years)# <50>/ = 50	160	52.0(31–75)					0.349
Tumor size (cm)$	160	2.53±1.21					0.928
Histological grade						0.233	0.003
I	39	12(30.8)	17(43.6)	7(17.9)	3(7.7)		
II	95	15(15.8)	38(40.0)	28(29.5)	14(14.7)		
III	26	4(15.4)	7(26.9)	7(26.9)	8(30.8)		
LN status						−0.003	0.966
Negative	86	16(18.6)	36(41.9)	18(20.9)	16(18.6)		
Positive	74	15(20.3)	26(35.1)	24(32.4)	9(12.2)		
pTNM stage						0.163	0.040
I	47	11(23.4)	18(38.3)	11(23.4)	7(14.9)		
II	80	16(20.0)	38(47.5)	15(18.8)	11(13.8)		
III–IV	33	4(12.1)	6(18.2)	16(48.5)	7(21.2)		
ER status						−0.421	<0.001
Negative	61	4(6.6)	20(32.8)	20(32.8)	17(27.9)		
Weak	30	7(23.3)	8(26.7)	8(26.7)	7(23.3)		
Moderate	24	5(20.8)	11(45.8)	7(29.2)	1(4.2)		
Strong	45	15(33.3)	23(51.1)	7(15.6)	0(0.0)		
HER-2 status						0.08	0.316
−/+	120	24(20.0)	48(40.0)	32(26.7)	16(13.3)		
++/+++	40	7(17.5)	14(35.0)	10(25.0)	9(22.5)		

* , *P* values were calculated by Spearman's Rank-Correlation test (n = 160).

# , Age: Expressed as median (range), F = 1.105, *P* = 0.349 (ANOVA test).

$ , Tumor size: Expressed as mean ± standard deviation, F = 0.153, *P* = 0.928 (ANOVA test).

### SDCBP expression in different mammary epithelial cells

**Figure 2:**
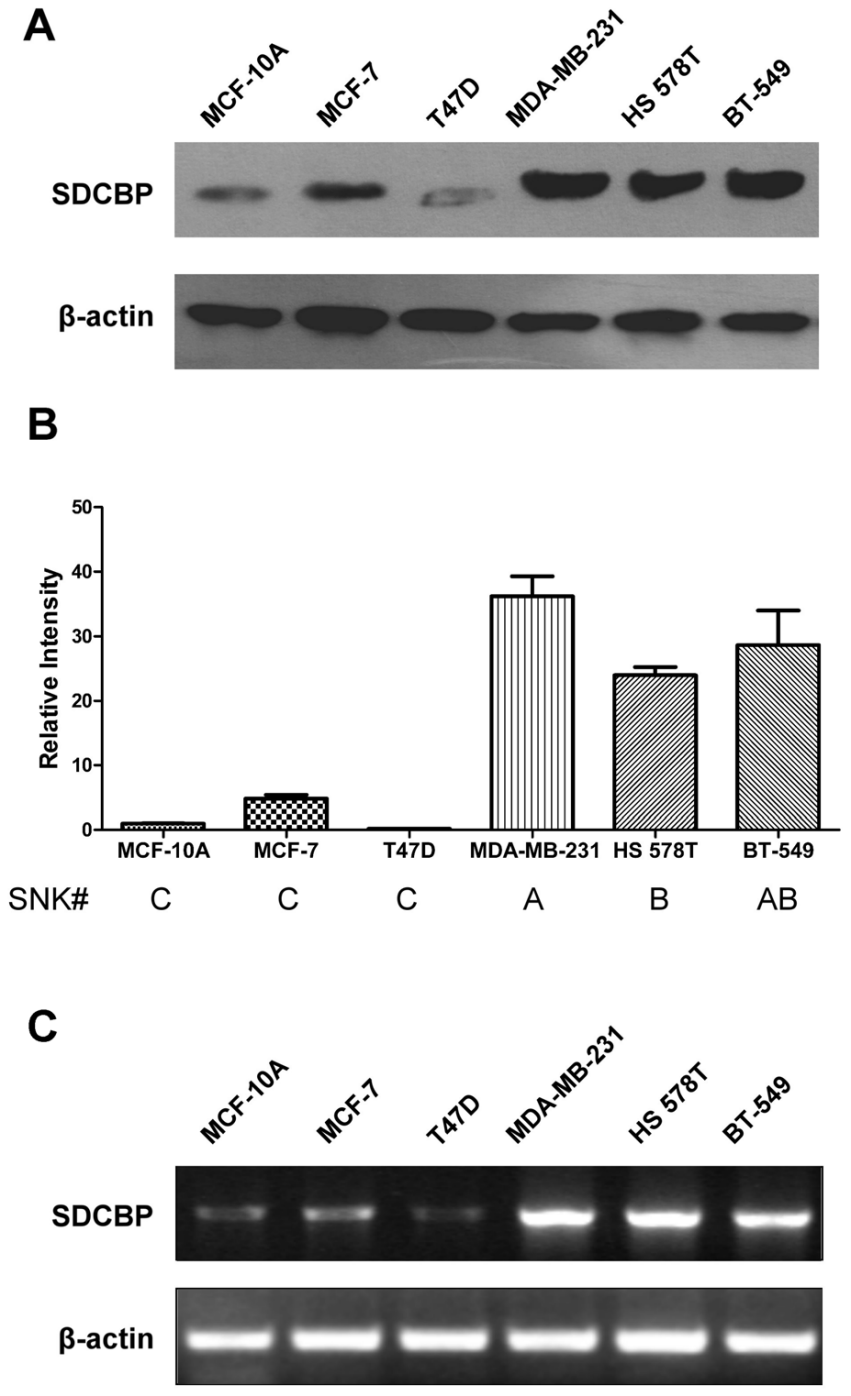
The expression profile of SDCBP in mammary epithelial cells. (**A**) Western Blot analysis of syndecan binding protein (SDCBP) in the breast fibrocystic disease epithelial cell MCF-10A, estrogen receptor (ER) positive breast cancer cells MCF-7 and T47D, as well as ER-negative MDA-MB-231, Hs 578T and BT-549. β-actin was used as the internal control. (**B**) The relative western-blotting intensity of SDCBP/β-actin from triplicate repeats of each cell line was calculated. SNK grouping test (*q* test) was used to compare the difference of SDCBP expression levels among these mammary epithelial cell lines, and (#) groups containing same letter(s) were not significantly different. (**C**) Semi-quantitative reverse transcription-PCR analysis of SDCBP in these cell lines. β-actin was used as the internal control.

Corrected by the relative intensity of SDCBP/β-actin, Western Blot of holoprotein from different mammary epithelial cell lines under regular culture conditions revealed that SDCBP protein expression was significantly higher in ER-negative MDA-MB-231, Hs 578T and BT-549 BCa cells than that in ER-positive MCF-7 and T47D cells and in the fibrocystic disease epithelial cell MCF-10A (Calculated by SNK grouping test; Figures 2A and 2B). It was also noticeable that between the two ER-positive cells lines, MCF-7 cells had higher expression of SDCBP than T47D cells.

Semi-quantitative RT-PCR analysis of the SDCBP expression revealed remarkably more mRNA accumulation in ER-negative MDA-MB-231, Hs 578T and BT-549 BCa cells than that in ER-positive MCF-7 and T47D cells and in the fibrocystic disease epithelial cell MCF-10A, which was consistent with the results on protein level (Figure 2C).

### SDCBP expression in the ER-positive MCF-7 cell line treated with E2

**Figure 3:**
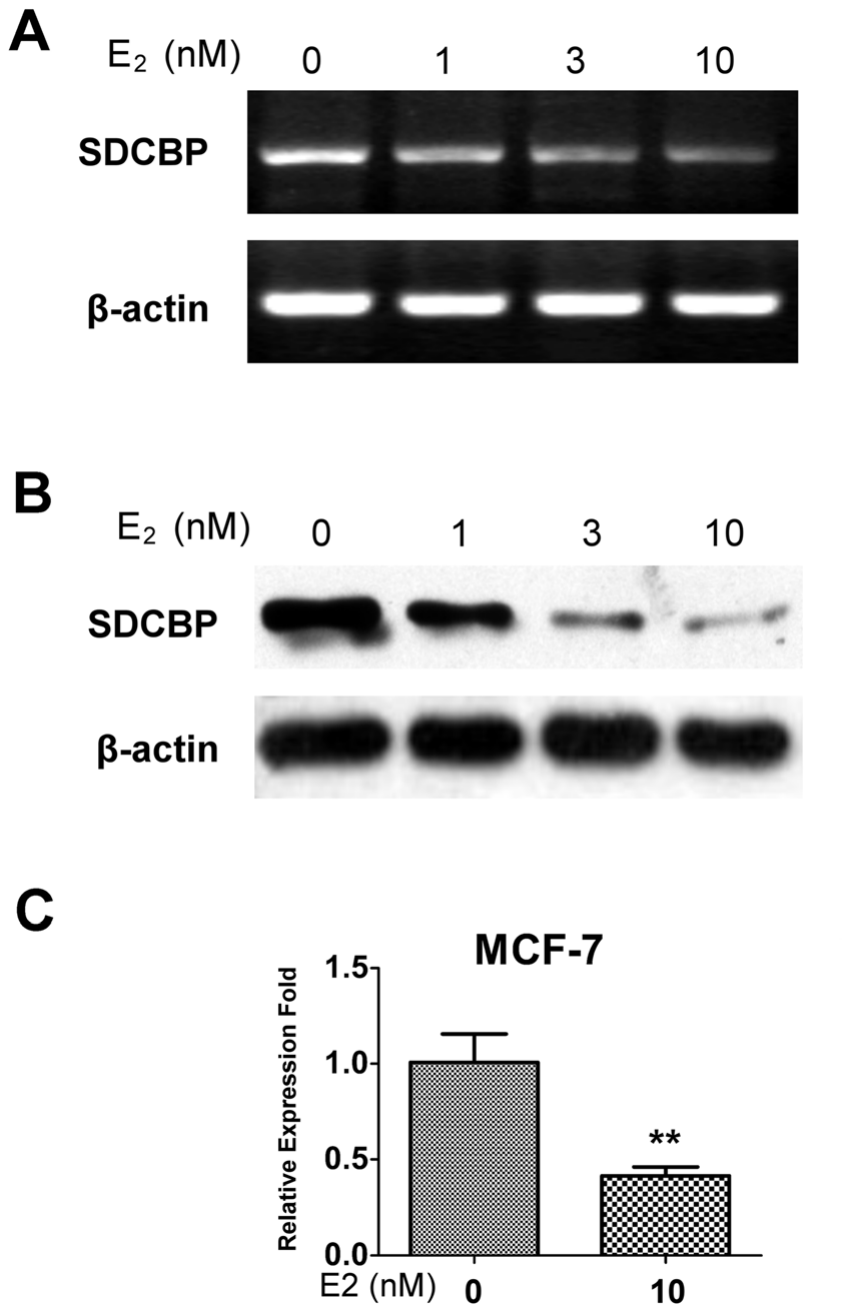
The relationship between SDCBP expression levels and estrogen treatment in estrogen-responsive MCF-7. (**A**) Semi-quantitative reverse transcription-PCR and (**B**) Western Blot analysis of syndecan binding protein (SDCBP) in MCF-7 cells under different concentrations of 17-β estradiol (E_2_) stimulation. β-actin was used as the internal control. (**C**) Quantitative analysis of SDCBP mRNA level in MCF-7 under steroid hormone deprivation and 10 nM E_2_ stimulation by real-time quantitative reverse transcription-PCR. SDCBP mRNA expression levels were normalized against β-actin mRNA expression. Each experiment was repeated three times. **P<0.01 (Student's *t*-test).

In the MCF-7 cells treated with E2, semi-quantitative RT-PCR and Western Blot analysis found SDCBP was down-regulated both at the mRNA and protein levels in a dose dependent manner (Figures 3A and 3B). qRT-PCR evaluation showed that 10 nM E2-treatment resulted in 58.8% down-regulation of SDCBP at mRNA level in MCF-7 cells (*P*<0.01; Figure 3C).

### SDCBP inhibition in BCa cells

**Figure 4:**
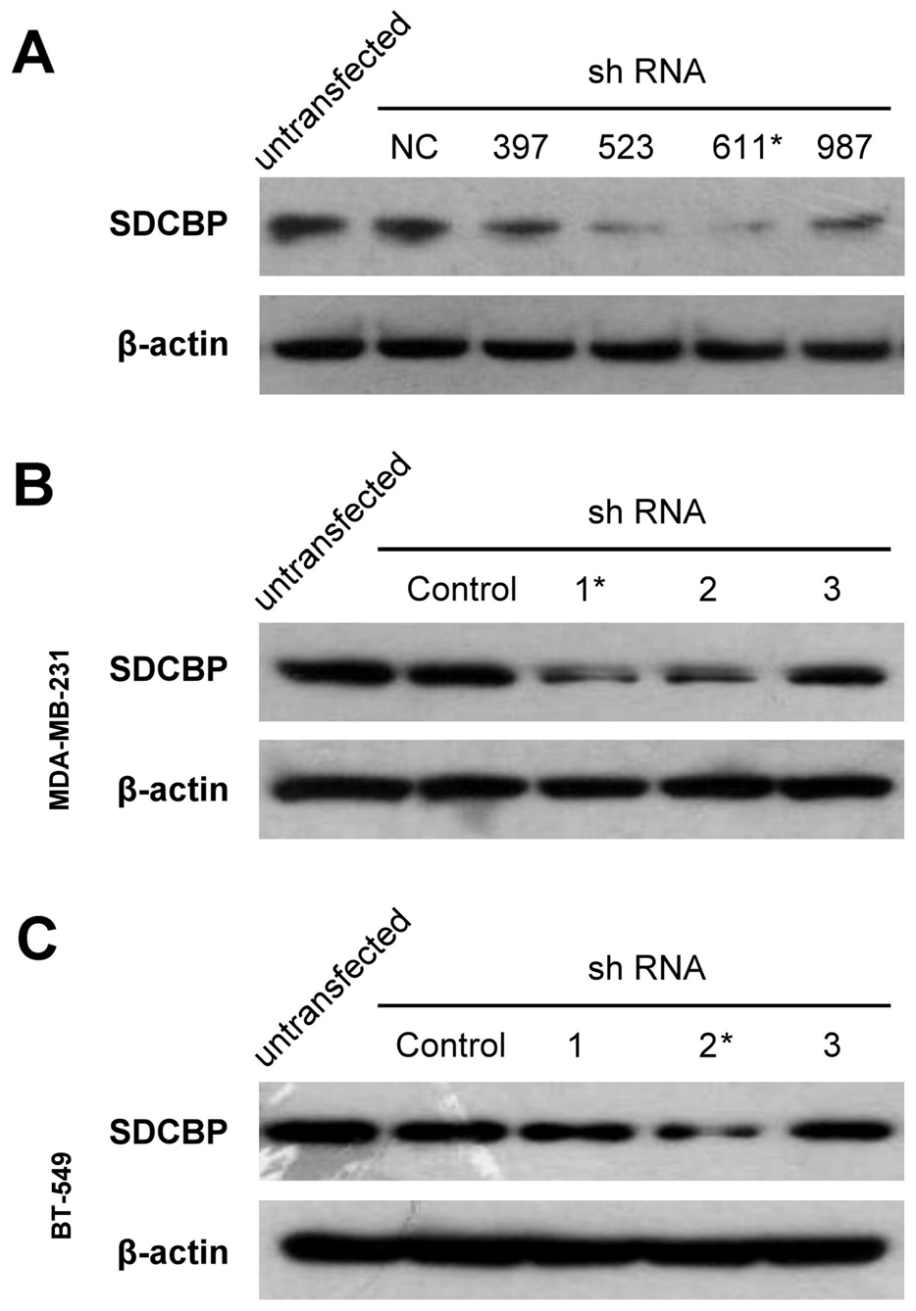
Construction of SDCBP silenced breast cancer cells. (**A**) Syndecan binding protein (SDCBP) and negative control (NC) short-hairpin RNA (shRNA) expression constructs were transiently transfected into human embryonic kidney 293T cells and the holoproteins in cell lysates were western-blotted for SDCBP shRNA selection. Digits 397, 523, 611 and 987 represent initiation site of four candidate target sequences selected in SDCBP mRNA (NM_001007067) and (*) represent the target sequence we selected as SDCBP shRNA. (**B**) MDA-MB-231 cells with SDCBP silenced were selected by Western Blot analysis. (*) represented the clone we selected as the MDA-MB-231-SDCBP shRNA cell. (**C**) BT-549 cells with SDCBP silenced were selected by Western Blot analysis. (*) represented the clone we selected as the BT-549-SDCBP shRNA cell.

The target sequence (GGGACCAAGTACTTCAGATCA, 611^#^) was selected as the final RNA interference sequence for SDCBP (Figure 4A). Clone 1 of MDA-MB-231 and Clone 2 of BT-549 were selected as SDCBP silenced cell clones by Western Blot for further research in the article respectively (Figures 4B and 4C).

### SDCBP silence and BCa cell proliferation *in vitro* and *in vivo*

**Figure 5:**
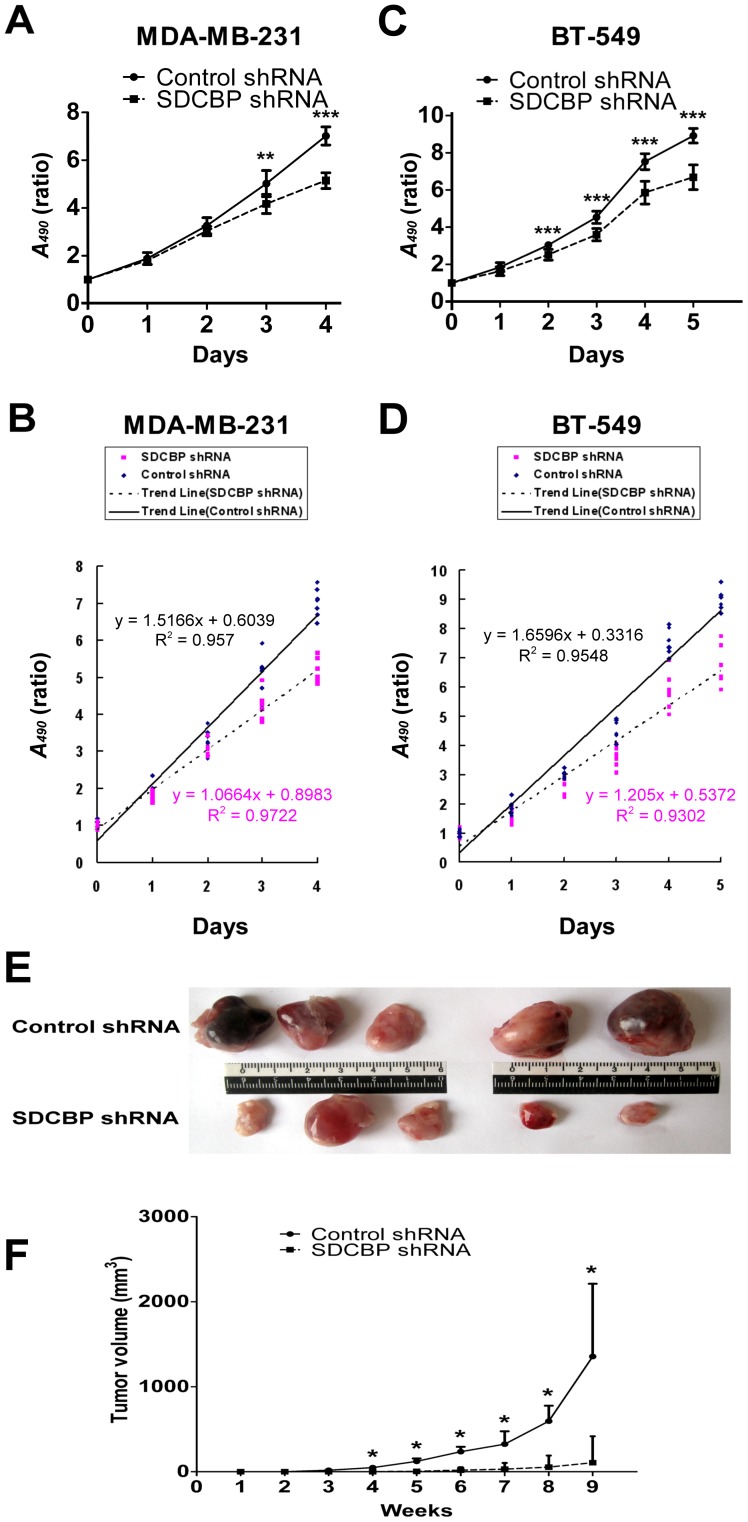
Effects of SDCBP silence on breast cancer cell growth *in vitro* and *in vivo*. (**A**) 3-(4, 5-dimethylthiazol-2-yl)-2,5-diphenylte-trazolium bromide (MTT) assays as well as (**B**) their corresponding linear regression models were performed to evaluate *in vitro* growth rate of MDA-MB-231-SDCBP shRNA and MDA-MB-231-Control shRNA cells. (**C**) MTT assays and (**D**) their corresponding linear regression models were performed to evaluate *in vitro* growth rate of BT-549-SDCBP shRNA and BT-549-Control shRNA cells. **P<0.01; ***P<0.001 (Student's *t*-test). (**E**) MDA-MB-231-SDCBP shRNA and MDA-MB-231-Control shRNA bearing tumors were removed from the corresponding inoculated mice respectively and compared after 9 weeks of inoculation. (**F**) Tumor volumes of these two groups were measured, calculated and compared at the end of each week. *P<0.05 (Mann–Whitney *U*-test).

Growth curve showed that SDCBP silence slowed down the growth of both MDA-MB-231 and BT-549 cells. At the end of “Days 4”, the *A_490_* ratio of MDA-MB-231-SDCBP shRNA cells was decreased by 26.6% in comparison with that of MDA-MB-231-Control shRNA cells (*P*<0.001; Figure 5A). Using SPSS 13.0 software, we performed linear regression analysis on *A_490_* ratio of both control and shRNA groups in the first 4 days. Two regression models for the relationship between “*A_490_* ratio” and “days” were acquired and these two fitted straight lines are statistically meaningful (MDA-MB-231-Control cells: y = 1.5166x+0.6039, R^2^ = 0.957, and *P*<0.001; MDA-MB-231-SDCBP shRNA cells: y = 1.0664x+0.8983, R^2^ = 0.9722, and *P*<0.001). Slope of SDCBP shRNA (95% CI: 1.003–1.130) was considerably lower than that of control shRNA (95% CI: 1.403–1.630) (Figure 5B). At the end of “Days 5”, the *A_490_* ratio of BT-549-SDCBP shRNA cells was decreased by 25.0% in comparison with that of BT-549-Control shRNA cells (*P*<0.001; Figures 5C). The linear regression analysis on models between “*A_490_* ratio” and “days” in the first 5 days also identified statistically meaningful (BT-549-Control shRNA cells: y = 1.6596x+0.3316, R^2^ = 0.9548 and *P*<0.001; BT-549-SDCBP shRNA cells: y = 1.205x+0.5372, R^2^ = 0.9302, and *P*<0.001). The slope of SDCBP shRNA group (95% CI: 1.100–1.310) was also remarkably lower than its control counterpart (95% CI: 1.544–1.775) (Figure 5D). The results indicated SDCBP silence inhibited ER-negative tumor cell growth *in vitro*.

All mice injected with MDA-MB-231 cells developed tumors (Figure 5E). Starting from the fourth week, the volumes of MDA-MB-231-SDCBP shRNA bearing tumors had been significantly smaller than those of MDA-MB-231-Control shRNA ones and after 9 weeks of inoculation, the median tumor volume of the former ones was 90% smaller than that of the latter ones. (*P*<0.05; Figure 5F).

### Effect of SDCBP silence on BCa cell cycle

**Figure 6:**
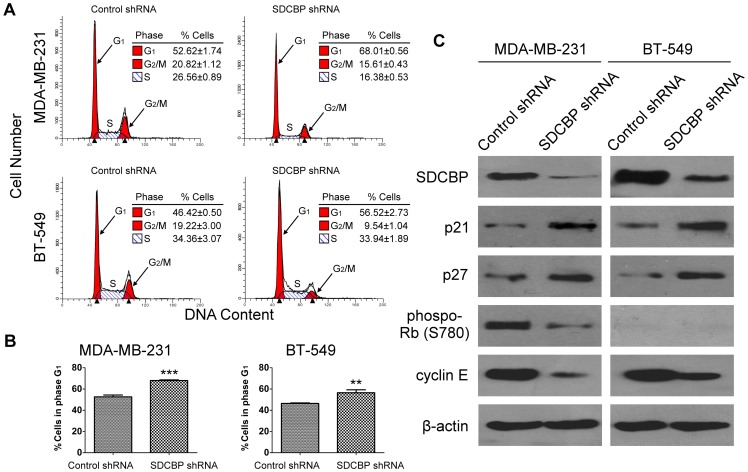
The effect of SDCBP silence on breast cancer cell cycle. (**A**) Flow cytometry were used to analyze the cell cycle. The proportion of cells at G_1_, S and G_2_/M phase were represented by three independent experiments. (**B**) The proportions of syndecan binding protein (SDCBP) silenced MDA-MB-231 and BT-549 cells at G_1_ were compared with those of their corresponding control shRNA transfected cells at G_1_ respectively. **P<0.01; ***P<0.001 (Student's *t*-test). (**C**) The cell cycle regulators p21, p27, phospo-Rb (S780) and cyclin E were western-blotted in above-mentioned cells. β-actin was used as the internal control.

Cell cycle of SDCBP silenced MDA-MB-231 and BT-549 cells as well as their controls were analyzed and cell proportions in different phases of cell cycle were compared (Figure 6A). We found SDCBP silence induced a 29.2% increase of MDA-MB-231 cells at G1 phase (*P*<0.001; Figure 6B). Similarly, 21.8% more BT-549 cells was identified in the G1 phase when SDCBP was silenced (*P*<0.01; Figure 6B). Corresponding to G1 accumulation, cells in S and G2/M phases were decreased in MDA-MB-231-SDCBP shRNA cells while only cells in G2/M but not in S phase decreased in BT-549-SDCBP shRNA cells (Figure 6A).

Cell cycle arrest induced by SDCBP silence was further investigated by exploring its effects on the expression of related critical cell cycle regulators: p21 and p27, phospo-Rb (S780), and the G1/S-checkpoint-related cyclin E. It was found that p21 and p27 were up-regulated and cyclin E was down-regulated in both SDCBP silenced cell lines. Phosphorylated Rb was down-regulated in MDA-MB-231-SDCBP shRNA cells, while its expression was neither identified in SDCBP-silenced BT-549 cell line nor in its control (Figure 6C).

## Discussion

In normal or breast carcinoma cells with functional ER, estrogen-signaling pathway is critical for cell proliferation [15]. Estrogen mediates the transition of cells from the G1 to S phase of the cell cycle [16]. However, BCa cells can also maintain growth and proliferation through other signaling pathways when estrogen-signaling pathway is not available due to complex regulation of gene expression. So far, we have only very limited knowledge of the potential alternative pathways. Here, SCDBP is investigated as a candidate marker that may participate in the alternative pathways to maintain the growth of BCa cells without functional ER.

In the first step, we did found that SDCBP was up-regulated in 160 BCa tissues, in comparison with 23 counterparts of normal breast tissues (*P*<0.001). Furthermore, its expression was negatively correlated with ER grading (R_S_ = −0.421, *P*<0.001): Overexpression was mainly identified in ER-negative tumors in comparison with that in ER-positive tumors. These results were suggestive that SDCBP expression was associated with the tumor behavior of ER-negative BCas and allowed us to sense that we were chasing around the appropriate target. SDCBP expression was also identified positively correlated with the histological grading (R_S_ = 0.233, *P* = 0.003) and pTNM stating (R_S_ = 0.163, *P* = 0.04) of BCa, indicating that its expression in tumor tissue may have prognostic significance,.although a positive association of its expression with tumor HER-2 overexpression was not significant in this cohort. Validation of these findings in a larger scale of tumor samples as well as acquirement of their corresponding follow-up information is required.

The same pattern of SDCBP expression was identified in mammary epithelial cell lines: its expression was remarkably higher in ER-negative MDA-MB-231, Hs 578T and BT-549 BCa cells than that in ER-positive MCF-7 and T47D cells and in the fibrocystic disease epithelial cell MCF-10A. Although MCF-7 had higher expression of SDCBP than T47D, their SDCBP expressions are both clearly lower than those of ER-negative cell lines, to a statistically significant extent. Koo et al reported that only a weak signal of SDCBP expression was detected in both MCF7 and T47D at protein level [6], which is more beneficial for our conclusion. The disparity between our data is probably due to the different source of fetal bovine serum (FBS) we used. When MCF-7 cells were treated with E2, a dose-dependent down-regulation of SDCBP was identified both at the mRNA and protein levels. So apart from the results of immunohistochemical staining, this is another piece of evidence to support the conclusion of the study that SDCBP expression is negatively correlated with the ER expression of the cells. These findings further support the assertion that SDCBP participates in the alternative pathways to maintain the growth of BCa cells when estrogen-signaling pathway is not available.

*In vitro* and *in vivo* studies on the SDCBP silence also provided additional evidences to support this view. The SDCBP silence slowed down the growth rate of both MDA-MB-231 and BT-549 cells. In the nude mice, the volumes of MDA-MB-231-SDCBP shRNA bearing tumors were significantly smaller than those of MDA-MB-231-Control shRNA ones.

The next question to ask is how SDCBP involves in the tumor cell proliferation of ER-negative BCa. To answer this question, we studied the tumor cell cycle changes when SDCBP was silenced in MDA-MB-231 and BT-549. In both cell lines with SDCBP suppression, we found a significant increase of cells at G1 phase, in contrast to those with intact SDCBP function. (*P*<0.001; *P*<0.01). In cell cycle regulation, the transition from G1 to S phase is the most commonly noted cell-cycle abnormality in tumors [17]. During normal cell cycle, cyclin E level is low at G1 phase and is increased during the transition from G1 to S phase. High levels of cyclin E correlate strongly with a poor outcome in patients with BCa [18]. Hence the SDCBP-silence-induced cell-cycle arrest was further investigated by exploring its effects on the expression of related critical cell cycle regulators including cyclin E. In both MDA-MB-231 and BT-549 cell lines, it was found that SDCBP silence inhibited cyclin E expression and recovered p21 and p27 expression. However, we did note a difference between the two cell lines, that is: SDCBP silence notably decreased the phosphorylation of Rb in MDA-MB-231, while phosphorylated Rb was negative in BT-549 due to the non-functional Rb gene of the cell line [19]. Zhang et al. found in their study that p27 could also reduce the cyclin E levels and mediate cell cycle arrest independent of Rb phosphorylation [20], which may explain the changes of cyclin E caused by SDCBP silence in BT-549. The evidence accumulated in the cell cycle study supported that SDCBP facilitated the BCa cells passing through the G1/S checkpoint and exerted its effect in cell proliferation by promoting cyclin E expression. Cell cycle analysis also showed a reduction of G2/M cells in both cell lines but no effect on S phase is observed in BT-549 cells. This disparity might due to other effect of SDCBP silence such as DNA replication delay and therefore S phase delay in BT-549, apart from preventing cell cycle through the G1/S checkpoint. In the SDCBP silenced BT-549 cells, the effect of decreasing cells in S phase as a result of increasing cells in G1 might be counteracted by the concurrent effect of S phase delay and its ensuing increase of cells in S phase. Therefore, there is no obvious change in the ratio of cells in S phase between BT-549-SDCBP shRNA and BT-549-Control shRNA cells.

Although both MDA-MB-231 and BT-549 are TNBC cell lines, their molecular characteristics are not entirely identical. For example, the BT-549 cells showed no expression of Rb [19] and pTEN [21], while MDA-MB-231 does. But the effects of SDCBP silence on G1 phase are similarly in both cells. These may be because that SDCBP was positioned in the upstream of multiple tumor-associated signaling pathways. SDCBP can interact with a variety of receptor tyrosine kinase, such as Eph family members [22]. It can also associate with the integrin-linked kinase (ILK) [23] and frizzled-7, a receptor for Wnt [24]. Through these interactions, SDCBP can activate other molecules, such us Akt/PKB [23] and c-jun [24], which may contribute to the progression of BCa. Li et al's study also suggested that SDCBP might regulate p53 by balancing the regulation of eIF5A signaling to p53 for apoptosis [5]. To understand the relationship between SDCBP expression and cell invasion/migration is also an interest of our laboratory and further exploration of the SDCBP-silence effect on apoptosis and metastasis of BCa will be expected.

In summary, our results suggest that SDCBP may function as an activator of alternative signaling pathways for cell proliferation of ER-negative BCa where the estrogen signaling pathway was not available. We speculate that specific suppression of SDCBP expression have potentials to become a targeted therapy for ER-negative BCa, and possibly including the TNBCs. Lack of SDCBP expression in normal breast tissues meets another key requirement for candidates of therapeutic targets.

## Supporting Information

Table S1 Antibody sources and work concentration.
(DOC)

Table S2 Primers for semi-quantitative and real-time quantitative reverse transcription-PCR.
(DOC)

Table S3 Candidate target sequence for short-hairpin RNA design of syndecan binding protein (SDCBP).
(DOC)
