# Supplementary material for: Syndecan Binding Protein (SDCBP) Is Overexpressed in Estrogen Receptor Negative Breast Cancers, and Is a Potential Promoter for Tumor Proliferation
Source: PLoS One. 2013 Mar 22;8(3):e60046. doi: 10.1371/journal.pone.0060046 (PMC3606191; doi:10.1371/journal.pone.0060046)
Supplement: Table S1 — Antibody sources and work concentration. (DOC) [file pone.0060046.s001.doc]

**Table S1. Antibody sources and work concentration.**

| **Antibody** | **Dilution** | **Source** |
| --- | --- | --- |
| Syndecan binding protein (SDCBP) | 1:75 | Abcam, USA |
| ER | 1:150 | Zymed, USA |
| HER-2 | 1:800 | Newmarker, USA |
