# Supplementary material for: Syndecan Binding Protein (SDCBP) Is Overexpressed in Estrogen Receptor Negative Breast Cancers, and Is a Potential Promoter for Tumor Proliferation
Source: PLoS One. 2013 Mar 22;8(3):e60046. doi: 10.1371/journal.pone.0060046 (PMC3606191; doi:10.1371/journal.pone.0060046)
Supplement: Table S2 — Primers for semi-quantitative and real-time quantitative reverse transcription-PCR. (DOC) [file pone.0060046.s002.doc]

**Table S2. Primers for semi-quantitative and real-time quantitative reverse transcription-PCR.**

| **Official Symbol of Target Gene** | **Genebank No.** | **Amplified Fragment Length (bp)** | **Annealing Temperature (℃)** | **Primer Name** | **Sequence (from 5’ to 3’)** |
| --- | --- | --- | --- | --- | --- |
| SDCBP | NM_001007067 | 106 | 55 | SDCBP foward | TGCTCCTATCCCTCACGATG |
|  |  |  |  | SDCBP reverse | GGCCACATTTGCACGTATTT |
| β-actin | NM_001101 | 194 | 55 | β-actin forward | GTCACCAACTGGGACGACAT |
|  |  |  |  | β-actin reverse | AGCACAGCCTGGATAGCAAC |
