# Supplementary material for: Syndecan Binding Protein (SDCBP) Is Overexpressed in Estrogen Receptor Negative Breast Cancers, and Is a Potential Promoter for Tumor Proliferation
Source: PLoS One. 2013 Mar 22;8(3):e60046. doi: 10.1371/journal.pone.0060046 (PMC3606191; doi:10.1371/journal.pone.0060046)
Supplement: Table S3 — Candidate target sequence for short-hairpin RNA design of syndecan binding protein (SDCBP). (DOC) [file pone.0060046.s003.doc]

**Table S3. Candidate target sequence for short-hairpin RNA design of syndecan binding protein.**

| **Target Site** | **Target Sequence (from 5’ to 3’)** |
| --- | --- |
| Negative control | AATTCTCCGAACGTGTCACGT |
| 397^#^ | GCAAGACCTTCCAGTATAAAC |
| 523^#^ | GGACTCAGGCTTAAATCAATA |
| 611^#^ | GGGACCAAGTACTTCAGATCA |
| 987^#^ | GCGGATGGCACCAAGCATTAT |

#: The numbers represents the position of the 5’ starting site of target sequences in syndecan binding protein mRNA (NM_001007067).
